# Skepticism and defiance: Assessing credibility and representations of science

**DOI:** 10.1371/journal.pone.0250823

**Published:** 2021-09-01

**Authors:** Jean Louis Tavani, Anthony Piermattéo, Grégory Lo Monaco, Sylvain Delouvée

**Affiliations:** 1 Laboratoire Parisien de Psychologie Sociale (EA 4368)—Groupe de Recherche sur la Parole et la Pensée Sociale—Université Paris 8 –Vincennes-Saint-Denis, Saint Denis, France; 2 Université Catholique de Lille, OCeS, Ethics (EA7446), Lille, France; 3 Aix Marseille Univ, ADEF–EA 4671, Marseille, France; 4 Univ Rennes, LP3C (Laboratoire de Psychologie: Cognition, Comportement, Communication)–EA 1285, Rennes, France; Medical University Innsbruck, AUSTRIA

## Abstract

Since the 1970s, there has been a growing interest in how individuals appropriate scientific knowledge, which has recently been reinforced by societal issues such as vaccine releases and skepticism about global warming. Faced with the health and social consequences of the mistrust of scientific knowledge, there is an urgent need for tools to measure the acceptance or rejection of scientific knowledge, while at the same time gaining a more detailed understanding of the processes involved. This is the purpose of this article. Thus, we conducted 4 empirical studies to provide a validation of the Credibility of Science Scale from the perspective of a French population, which aims to assess the credibility that individuals attribute to science and to empirically evaluate the link that may exist between the different levels of credibility attributed to science and the social representations of science. Studies 1a and 1b demonstrated good structural validity, the good fidelity (homogeneity and temporal stability), and the good criterion validity of the French version of the scale. In Study 2, we observed the same psychometric qualities of the French version of the scale. We also noted a structuring of the social representation of science based on age (Factor 1) and on the credibility attributed to science (Factor 2). Our results also raise the question of possible means of intervention to promote a better perception of science.

## Introduction

In 2019, the World Health Organization identified mistrust of vaccines as one of its ten priority struggles. In the same year, the Director-General of the World Health Organization declared that rumors and misinformation regarding vaccines, spread mainly on the Internet, constituted a bigger threat than the diseases themselves [1]. One illustration of the detrimental consequences of this phenomenon is the outbreaks of measles occurring all over the world, including countries where this virus was supposed to be have been eliminated, such as the U.S.A. Such a phenomenon can indeed be attributed to misinformation from anti-vaccine groups but also to a lack of trust in public health agencies [2,3]. As such, the case of vaccine hesitancy is just one example of a larger phenomenon: as public opinion tends to be increasingly suspicious of scientific studies and scientists, accusing them of hiding the truth and being influenced by lobbies, the credibility of science is becoming a major issue [4]. Indeed, science seems to be one of the most controversial issues in public discourse and in the media. Bauer [5] notes that there is "a crisis of trust. . . a decline in public confidence in science". However, as demonstrated by the example of vaccine hesitancy, this mistrust of science is not without consequences, especially if we consider, on the one hand, the pressure that suspicious public opinion can exert on the development of public policies (based on scientific data), and on the other hand, the influence of such a positioning on the adopted behaviors. In this context, aside from vaccine hesitancy [6,7] it is also possible to mention the impact that this mistrust of science (and institutions more generally) can have regarding global warming [8]. From different objects, Rutjens & van der Lee [9] show that science skepticism is on the rise in secularized countries.

In addition to these dynamics of contestation of science, there is a growing adherence to conspiracy beliefs and fake news [10,11]. Thus, attitudes towards science are polarized and oscillate between boundless trust in and complete rejection of scientific knowledge [4]. As these phenomena are part of a general dynamic, which emerged in the 1970s [12,13], and has not spared Europe [5,14], having a valid tool to measure them and highlight these main correlates in a French-speaking context seems important. Indeed, in France, one in three individuals does not believe in the safety of vaccines; thus France is one of the most skeptical countries [15]. In a more recent survey on "French confidence in science" [16] it is noted that despite a high level of confidence in science, doubts exist about its use in the public space. Finally, in a study conducted in September 2019 in France, on a sample of more than 2,000 individuals, representative of the French population aged 18 and over, scientists remained the most trusted of the professions, behind firefighters, nurses, and doctors. The trust placed in them has been relatively stable since 2013. The current pandemic perfectly illustrates this ambivalence of the French population towards the vaccine. If Detoc et al [17] in a survey conducted in April 2020 show that 75% of French people are ready to be vaccinated against Covid-19, Bertin et al [18] point out that a conspiracy mentality negatively predicts participants’ intentions to be vaccinated against VIDC-19 in the future. While the first vaccines are available,the French government has been afraid of anti-vaxxers and the public mistrust [19].

From this perspective, the objective of this article is to provide a validation in the French context of the Credibility of Science Scale (CoSS), which aims to assess the credibility that individuals attribute to science [20], and to empirically highlight the link that may exist between the different levels of credibility attributed to science and the social representations of science, referring to knowledge, beliefs and socially constructed opinions about this object. Although there is a tradition of research into the social representations of science, to our knowledge they have never been studied in relation to the credibility of science. To do this, we have carried out three studies. The first two aim to study the psychometric qualities of this tool and the third aims to replicate some of these results and test the validity of the scale’s criteria by exploring its possible links with social representations of science.

### Perception of science

When researchers are interested in the issue of perceived credibility of science, they are interested in inter-individual or inter-group differences arising from the issuer of the information (e.g., a scientist’s facial appearance [21]; the properties of the message itself [22]; and/or the characteristics of the audience receiving the message. Gauchat [23] studying temporal trends in public trust in science in the United States between 1974 and 2010 show differences by social class, ethnicity, gender, church attendance, region and especially political conservatism. Donald Trump, and more broadly anti-intellectual attitudes, have accelerated distrust and mistrust of scientists and science. [24,25]. Here we focus on the audience, and more specifically on the attitude that individuals may have towards science [22], but also on the social representations they may have of the same object.

### Social representations and the representation of science

Social representations can be defined as “systems of opinions, knowledge, and beliefs” particular to a culture, a social category, or a group with regard to objects in the social environment” [26]. However, this theory is not limited to observing how individuals and groups communicate about social objects, but also aims to understand how information, and sometimes information from science, can be reconstructed and play a functional role in controlling and understanding the environment. Indeed, the theory of social representations has from the outset had the objective of considering the permeability that can exist between scientific knowledge and knowledge of common sense (cf. "lay thinking") [27,28]. From such a perspective, Moscovici’s [27] early work on psychoanalysis proposed viewing social representations as socio-cognitive structures aimed at understanding how common sense appropriates a scientific theory. This is how scientific information passes through the filter of common sense and via a system of reciprocal exchange, the science represented becomes controlled, usable and mobilizable. It is in this perspective that certain diseases with well-known etiology are attributed causes of transmission or become contagious when they are not (e.g., AIDS) in order to serve very specific social exclusion issues (e.g., discrimination against homosexuals).

The theory of social representations makes it possible to go beyond the idea that common-sense knowledge about science should be interpreted as a deviation from a rational, objective knowledge of science and to assume that the ignorance of individuals and groups can account for behaviors or attitudes towards that object [29], and in particular the hypothesis that a lack of knowledge of science and its methodology would lead inexorably to its rejection [11,30]. Indeed, the theory of social representations assumes that deviations from objective, formal, or rational thinking are based on a logic that is specific to the group [31]. Thus, by making sense of these gaps, behaviors and attitudes can be explained. Indeed, several studies have highlighted a link between social representations and not only behaviors [32], the legitimization of the latter [33], but also attitudes [34,35]. Thus, taking into account the representations of science would make it possible to highlight the potentially causal links between these representations and, on the one hand, attitudes towards science and, on the other hand, the various preventive or health-promoting behaviors, but also the justification that individuals may have for the latter. The use of studies on the social representation of science thus makes it possible to go beyond the study of attitudes by placing the latter in the context of intergroup relations and by offering the possibility of qualitative and quantitative indicators of the way in which individuals make sense of science and scientists [30].

More specifically, studies on the social representation of science have shown that in France, there are two social representations of science, one popular, corresponding to a vision of science as an ill-defined and relatively broad field, and one more specific to people with a certain scientific culture for whom science is a well-defined domain [29]. These results can be found in other countries and, as such, these representations will have an impact on the judgement of the scientificity of certain disciplines. Thus, biology is seen as more scientific for those who see science as an ill-defined field. In fact, for the latter, the representation of science is articulated around the medical sciences [12,29]. Thus, the way medicine is represented will impact the whole representation of science, and as such science can be characterized by the specific attributes of medicine: it is an applied science, these applications are seen as beneficial, and researchers working in this field are also clinicians who will apply their scientific work. Science would therefore be seen through the prism of its social utility [29]. Work on social representations of science has also shown that trust in science is linked on the one hand to the competence attribute to scientists and on the other hand to the disinterestedness attributed to scientists. [12]. Since the 1970s, various scandals have undermined the disinterestedness of scientists. People increasingly see scientists as having hidden motives and as playing into the hands of various private organizations, which are also increasingly funding scientific research [4,12].

### The Credibility of Science Scale (CoSS)

The CoSS is composed of 6 items in the form of statements (see Table 1). For each item, participants give their level of agreement on a 7-point semantic scale (1. Disagree very strongly, 2. Disagree strongly, 3. Disagree somewhat, 4. Neither agree nor disagree, 5. Agree somewhat, 6. Agree strongly, 7. Agree very strongly). All items were translated into French and a back-translation process was used to check the translation [36,37]. The original version of the scale shows that when individuals strongly adhere to a religion, or oppose governments, they will also show more mistrust of science [20]. In addition, individuals who strongly adhere to conspiracy beliefs will also be more suspicious of science, or reject it [20,38]. Finally, the scores obtained using the science credibility scale correlate with the openness dimension in the Big Five personality factor model.

**Table 1:** Descriptive statistics of the items on the CoSS–Study 1a.

	M	SD	Min	Max	Skewness	Kurtosis
People trust scientists a lot more than they should	4.31	1.61	1.00	7.00	-0.20	-0.82
People don’t realize just how flawed a lot of scientific research really is	4.97	1.41	1.00	7.00	-0.61	0.03
A lot of scientific theories are dead wrong	4.10	1.55	1.00	7.00	0.02	-0.54
Sometimes I think we put too much faith in science	4.45	1.72	1.00	7.00	-0.41	-0.79
Our society places too much emphasis on science	3.77	1.69	1.00	7.00	0.11	-0.92
I am concerned by the amount of influence that scientists have in society	3.69	1.69	1.00	7.00	0.15	-0.81
CoSS	4.22	1.14	1.00	7.00	-0.14	-0.24

### Ethics statement

This project has been approved by the (anonymized link) Ethics Committee approval number: 2020–002. We thus applied the 1964 Helsinki declaration and its later amendments [39], the ethical principles of the French Code of Ethics for Psychologists [40], and the American Psychological Association Ethical Principles of Psychologists and Code of Conduct [41]. Participants were informed about the purpose of the study in a cover letter and were assured that their data would remain confidential. Agreement to participate by completing and returning the questionnaire was taken as consent. All research materials were uploaded to the Open Science Framework (https://osf.io/32b4x/?view_only=6934c8ff547b409384c025e8f0237b73).

## Overview of the current research

The objective of this article is twofold: to validate within a French population a scale designed to assess the credibility that individuals attribute to science and to highlight the fact that different levels of credibility towards science are associated with different social representations. To do this, we carried out three studies. The first two aim to study the psychometric qualities of the scale and the third aims to replicate some of these results and to test the validity of the scale’s criteria by exploring its possible links with social representations of science.

## Study 1a

The objective of this first study was to test the factor structure of the scale. The original English items were translated into French: each item was first translated by a native speaker of French and then back translated into English. Inconsistencies between the original and the back translated version and their implications for the translated version were resolved by discussion between the authors [42] (See Appendix 1 in S1 File, in additional material for the French version of the scale).

### Method

#### Participants and procedure

The study took the form of an online questionnaire distributed via social networks (on local self-help groups and local community groups). Data collection took place between 10 and 15 February 2019. The participants were invited by a message published in city self-help Facebook groups. The invitation message mentioned that the study was about how to see the world, with no specific mention of science. In order to determine the necessary sample size, we followed the recommendations to use a sample of at least 250 participants [43]. Four hundred and fifty-two participants had completed the questionnaire (*M*_*age* =_ 27.01; *SD =* 7.94, age range: 18–62). 59 participants reported that they were distracted or disturbed during the questionnaire, so they were excluded from the analysis. The final sample consisted of 393 participants (*M*_*age* =_ 27.18; *SD =* 8.02, age range: 18–58 year; 90.84% of women). After freely consenting to participate in the study, participants completed some socio-demographic characteristics (gender and age) and then completed the science’s credibility scale, whose items were presented in random order.

*Credibility of science scale*. The original scale consists of 6 items (see Table 1), which take the form of statements. Participants must say how much they agree with each other by giving their answers on a 7-point scale ranging from 1 (*very strongly disagree*) to 7 (*very strongly agree*). The analyses referring to the examination of psychometric qualities are presented in the results section.

### Results & discussion

The descriptive statistics of the different items indicated a relatively normal distribution of scores (see Table 1). A joint examination of the descriptive statistics of the overall score, the histogram representing the distribution of scores on the Science Credibility scale and the quantile-quantile plot of scores on the same scale (see additional material, Figs 1 and 2 in S1 File), indicated that the distribution was also relatively normal.

We performed a Principal Component Analysis (PCA) on the 6 items of the scale (KMO = .83; KMO per item > .79; χ^2^(15) = 655.06 p < .001). The PCA, based on a parallel analysis [44], highlighted only one component (λ = 3.01) to be extracted, which explained 50.22% of the variance. All item saturations on this single component were greater than .53.

We conducted a Confirmatory Factor Analysis (CFA) to test the one-dimensional factor structure predicted by the original work. We interpreted the fit indices based on the benchmark values found in recent literature [45–47], namely: Root Mean Square Error of Approximation (RMSEA; good if < .06, acceptable if < .08); the Standardized Root Mean Square Residual (SRMR; good if < .05, acceptable if < .08), and the Comparative Fit Index (CFI; good if > .95, acceptable if > .90), and the Tucker-Lewis Index (TLI; good if > .95, acceptable if > .90). The Confirmatory Factor Analysis indicated a good fit of the data to a one factor model (CFI = .963; TLI = .938; SRMR = .039; RMSEA = .082). Nevertheless, the value of the RMSEA was slightly too high, and examination of the residue correlation matrix told us to add a correlation between the errors of items 2 and 3, so the adjustment improved (CFI = .992; TLI = .985; SRMR = .020; RMSEA = .040). Finally, the internal consistency was adequate (α = .80). The item scores were averaged to obtain a total score that represented the credibility that participants attribute to science. Thus, the higher the score, the more credibility participants attribute to science.

The credibility score attributed to science correlated significantly with age (*r =* -.11, *p =* 0.03): the older people were, the less credibility they gave to science. However, we did not observe any significant difference according to the sex of the participants (*p =* 0.17).

This study gives us initial results indicating that some psychometric qualities of the scale are satisfactory, such as structural validity and homogeneity of the measurement. In addition, it provides a normal distribution of scores. We then conducted a second study to replicate these results and examine the validity of the measurement criteria.

## Study 1b

The objective of this study was to replicate the results of Study 1a and to extend them by looking at the temporal stability of the measurement and the criterion validity of the scale. To do this, we compared the scores on the scale of attitude towards science with the personality scores of the participants, as well as with their scores for conspiracy beliefs, objectivity, and finally their subjective social class. Indeed, previous studies have found positive correlations with the openness dimension of personality [20]. Attitude towards science is negatively associated with adherence to conspiracy beliefs [4,20] and correlates negatively with the level of education [20]. Thus, we have used here the subjective social class, which partly covers the level of education [48].

### Method

#### Participants, procedure & measures

The study took the form of an online questionnaire distributed via social networks (on local self-help groups and local community groups). Data collection took place between 20 November and 31 December 2018. The participants were invited by a message published in city self-help Facebook groups. The invitation message mentioned that the study was about how to see the world, with no specific mention of science. In order to determine the necessary sample size, we followed the same recommendations as before [43]. Thus, three hundred and forty-three participants completed the questionnaire (*M*_*age* =_ 37.43; *SD =* 12.57, age range: 18–74). 41 participants reported that they were distracted or disturbed during the questionnaire, so they were excluded from the analysis. The final sample consisted of 302 participants (*M*_*age* =_ 37.51; *SD =* 12.68, age range: 18–74; 85.10% of women). After freely consenting to participate in the study, participants completed some socio-demographic characteristics, then completed scales to measure their personality, their perception of objectivity, their adherence to conspiracy beliefs, and finally the credibility scale of science. For all scales used, the items were presented in random order.

Of this first sample, 165 participants left a valid e-mail address in order to participate in the second phase of the study (corresponding to a retest to study the stability of the scale). They were asked to complete a second questionnaire 15 days after completing the first. Thus, 96 completed the second phase (*M*_*age* =_ 33.55 years; *SD =* 11.94, age range: 18–65; 83.33% of women). On average, they completed the second questionnaire 38.76 days after the first test (*SD* = 14.67, time range: 17–69 days). Participants in this sample completed the CoSS only.

#### Measures

*Personality*. To assess the personality of participants, we used the French version [49,50] of the Ten Item Personality Inventory [51] to obtain a score for each of the five personality dimensions based on the Big Five model [52,53].

*Perception of objectivity*. Participants completed an objectivity scale consisting of 4 items [54,55]. Objectivity refers to the view that individuals believe that they are unbiased and that they can effectively free themselves from the effects of undesirable influences that might influence their judgements [54,55]. Each item takes the form of statements and participants were asked to rate their level of agreement on a 7-point scale ranging from 1 (*Strongly Disagree*) to 7 (*Strongly Agree*). After performing a PCA on these 4 items (KMO = .68 KMO per item > .64; χ^2^(6) = 297.25, *p* < .001), we performed a confirmatory factorial analysis. The first highlighted only one component to be extracted, based on a parallel analysis [44]. This single component (λ = 2.28) explained 57.00% of the variance. All item saturations on this single component were greater than 0.68. We sought to confirm this one-dimensional structure using a Confirmatory Factor Analysis. The analysis indicated a satisfactory adequacy of the data to a one-factor model (CFI = .915; TLI = .745; SRMR = .047; RMSEA = .203). Nevertheless, the value of the RMSEA was too high and that of the TLI was too low, the examination of the correlation matrix of the residues told us to add a correlation between the errors of items 2 and 3, so that the adjustment improved (CFI = .965; TLI = .791; SRMR = .027; RMSEA = .184), but they still did not indicate a satisfactory adequacy. Nevertheless, the internal consistency of the scale was satisfactory (α = .75). The item scores were averaged to obtain a total score that represented the perception of objectivity. The higher the score, the more objective the participants think they are.

*Conspiracy Beliefs*. As in the original study by Hartman and al. [20], participants completed a 15-item scale to measure conspiracy beliefs (Generic Conspiracist Beliefs) [56]. Each item takes the form of statements and participants were asked how true they thought they were on a 4-point scale from 1 (*certainly not true*) to 5 (*certainly true*). Although it has already been used in studies in a French-speaking context [57], it has not been the subject of a true psychometric validation. Thus, we will present some elements aimed at testing its adaptation to the Francophone context. First, we performed a PCA on these 15 items (KMO = .94 KMO per item > .87; χ^2^(105) = 2358.56, *p* < .001). A parallel analysis [44] indicated that only one component (λ = 7.32) should be used, which explained 48.80% of the variance. All item saturations on this single component were greater than .60. In the first instance, we conducted a Confirmatory Factor Analysis by indicating a theoretical model with a single latent variable. The analysis indicated an unsatisfactory adequacy of the data to this model (CFI = .850; TLI = .826; SRMR = .063; RMSEA = .113). Examination of the residue correlation matrix indicated that we should add correlations between the errors of items 3 and 8, between items 3 and 13 and between items 13 and 8 (these three items are the only ones that refer to aliens and/or UFOs), so that the adjustment improved considerably (CFI = .957; TLI = .948; SRMR = .039; RMSEA = .061). The internal consistency of the scale was satisfactory (α = .92). The item scores were averaged to obtain a total score that represented adherence to conspiracy beliefs. The higher the score, the more participants adhered to these beliefs.

*Subjective social class*. Individuals had to place themselves on a visio-analogical scale representing society [58]. The left side of the scale represented individuals with the lowest levels of income and education as well as the least prestigious jobs. The right side of the scale represented individuals with the highest levels of income and education and the most prestigious jobs. By default, the cursor was placed in the center of the scale, and participants were asked to position it according to their perception of their status.

*Credibility of Science Scale*. Finally, the questionnaire also included the 6 items of the CoSS presented in Study 1a (see above).

### Results & discussion

In terms of the psychometric qualities of the credibility scale with respect to science, the results of this study were similar to those observed in Study 1a. The descriptive statistics of the different items indicated a relatively normal distribution of scores (see Table 2). The joint examination of the descriptive statistics of the overall score (see Table 2), the histogram representing the distribution of scores on the Credibility of Science Scale, and the quantile-quantile plot of scores on the same scale (see additional material, Figs 3 and 4 in S1 File), indicated that the distribution was also relatively normal.

**Table 2:** Descriptive statistics of the items on the CoSS–Study 1b.

	M	SD	Min	Max	Skewness	Kurtosis
People trust scientists a lot more than they should	3.88	1.67	1.00	7.00	-0.02	-0.91
People don’t realize just how flawed a lot of scientific research really is	4.60	1.43	1.00	7.00	-0.47	-0.20
A lot of scientific theories are dead wrong	3.58	1.55	1.00	7.00	0.08	-0.71
Sometimes I think we put too much faith in science	3.76	1.73	1.00	7.00	-0.02	-1.03
Our society places too much emphasis on science	3.38	1.67	1.00	7.00	0.37	-0.77
I am concerned by the amount of influence that scientists have in society	3.37	1.69	1.00	7.00	0.37	-0.77
*CoSS*	3.76	1.27	1.00	7.00	0.11	-0.52

We carried out a PCA on the 6 items of the scale (KMO = .87 KMO per item >.84; χ^2^(15) = 872.92, *p* < .001). Parallel analysis [44] indicated that only one component (λ = 3.68) should be used, which explained 61.42% of the variance. All item saturations on this single component were greater than .66. A Confirmatory Factor Analysis indicated a correct fit of the data to a one-factor model (CFI = .968; TLI = .946; SRMR = .035; RMSEA = .101). Nevertheless, the value of the RMSEA was too high, and an examination of the residue correlation matrix told us to add a correlation between the errors of items 2 and 3, thus the adjustment improved (CFI = .979; TLI = .961; SRMR = .025; RMSEA = .086). Finally, the internal consistency was satisfactory (α = .83). The results of the analyses carried out on the re-test were comparable: the PCA highlighted a single KMO component = .89 KMO per item >.87; χ^2^(15) = 346.63, *p* < .001; λ = 4.03) which explained 67.12% of variance. The factor solution from the data from the first round had good adjustment indices (CFI = .998; TLI = .996; SRMR = .021; RMSEA = .027). The internal consistency was also satisfactory (α = .90). The item scores were averaged to obtain a total score that represented the credibility that participants gave to science. The higher the score, the more credibility participants gave to science. The temporal stability of the measurement (e.g., aspect of reliability) was also satisfactory (*r* = .77, *p* < .001). If we try to predict the scores of the second handover with those of the first, taking into account the time between the two, the effect of the latter is not significant (*B* = -0.00, *t* = -0.20, *p* = 0.84).

An analysis of correlations highlighted two significant correlations (cf. Table 3), the first, rather strong with the adherence to a conspiracy mentality and the second, weaker, with the subjective social class. Thus, the more participants adhere to conspiracy beliefs, the more they doubt the credibility of science. Finally, the more participants perceive themselves as coming from a high social status, the more confidence they have in science. In line with Hartman et al.’s results, we did not observe any significant difference according to the sex of the participants (*p =* 0.87).

**Table 3:** Correlations between the different variables presented in Study 1b.

		*r*	*p*
Conspiracy Beliefs		**0.56**	**< .001**
Objectivity		-0.01	.856
Personality—Big Five			
	Openness	-0.05	.349
	Consciousness	0.01	.881
	Extroversion	0.06	.319
	Agreeability	0.00	.964
	Emotional Stability	-0.06	.265
Subjective social class		**-0.16**	**.006**
Age		-0.03	.591

Note. Significant correlation coefficients are presented in bold.

We performed a regression analysis to predict the credibility given to science with the following variables: adhesion to conspiracy beliefs, Big five dimensions, subjective social class, and age (R^2^ = 0.32, *F*(9,290) = 15.41, *p* < .001). Results are presented in Table 4. Only the adhesion to conspiracy beliefs predicts positively the credibility accorded to science: the more participants adhere to conspiracy beliefs, the more distrustful they are of science.

**Table 4:** Regression analysis to predict credibility towards science (Study 1B).

Predictor	β	SE	95% IC	*t*	*p*
Intercept	1.81	0.64	[1.005;3.531]	3.53	< .001
Conspiracy Beliefs	0.84	0.07	[0.692;0.988]	11.16	< .001
Objectivity	-0.05	0.07	[-0.195;0.097]	-0.66	0.510
Openness	-0.08	0.05	[-0.180;0.012]	-1.73	0.085
Consciousness	-0.03	0.05	[-0.127;0.067]	-0.61	0.545
Extroversion	0.02	0.05	[-0.069;0.115]	0.50	0.620
Agreeability	0.00	0.06	[-0.125;0.116]	-0.07	0.943
Emotional Stability	-0.01	0.04	[-0.100;0.077]	-0.26	0.795
Subjective social class	0.00	0.04	[-0.075;0.083]	0.09	0.925
Age	0.00	0.01	[0.011;0.009]	-0.18	0.853

The psychometric qualities of the scale were satisfactory. Beyond a normal distribution of scores, the French version of the scale showed good structural validity, good fidelity (homogeneity and temporal stability), and good criterion validity. In accordance with the results of previous work [20] we found a positive, and rather strong, correlation between adherence to a conspiracy mentality and the credibility of science. However, we did not find the average correlation between the scale of credibility of science and the openness dimension of the Big Five. Participants’ perception of objectivity was also not related to the credibility given to science. Furthermore, an interesting result shows that the more participants perceive themselves to be from a high social class, the more credibility they give to science.

## Study 1c

The objective of this study is to replicate and extend the results of previous studies (studies 1a and 1b). In order to do so, we have introduced other measures that can be related to the perception of credibility of science. Thus, in addition to the measures of conspiracy beliefs, subjective social class, we added measures to assess the intention to be vaccinated against COVID-19, the perception of vaccine efficacy, and the political orientation. Moreover, in order to have a true measure of participants’ level of education—contrary to the one used in study 1b—we also added a measure of the highest level of education obtained by the participants. As we mentioned earlier, previous work has shown negative links between the credibility of science and conspiracy beliefs and positive links with the level of education [1,2]. Moreover, the more participants believe they can trust science, the less they reject vaccination [1,2].

### Method

#### Participants, procedure & measures

The study took the form of an online questionnaire distributed via social networks (on local self-help groups and local community groups). Data collection took place between 18 and 24 September 2020. The participants were invited by a message published in city self-help Facebook groups. The invitation message mentioned that the study was about how to see the world, with no specific mention of science. In order to determine the necessary sample size, we followed the recommendations to use a sample of at least 250 participants [43]. Three hundred and sixty-seven participants had completed the questionnaire (*M*_*age* =_ 27.75; *SD =* 11.42, age range: 18–72). 47 participants reported that they were distracted or disturbed during the questionnaire, so they were excluded from the analysis. The final sample consisted of 320 participants (*M*_*age* =_ 28.06; *SD =* 11.63, age range: 18–72 year; 78.12% of women). After freely consenting to participate in the study, participants completed some socio-demographic characteristics (age, gender, and their highest level of education), then completed scales to measure their intentions to be vaccinated against COVID-19, their perception of the effectiveness of vaccines in general, the CoSS, their adherence to conspiracy beliefs, and finally two measures to assess their subjective social class and political orientation. For all scales used, the items were presented in random order.

#### Measures

*Intentions to be vaccinated against COVID-19*. We used a single item to assess vaccination intentions against Covid-19: “Imagine that a vaccine against Covid-19 is developed, validated by health authorities and marketed. If you had the opportunity to be vaccinated against Covid-19 next week, what would you decide? Please give your answer on the following scale from”. Participants were asked to give their answers on a visio-analogical scale ranging from "0—I would refuse without any hesitation" to "10—I would accept without any hesitation".

*Usefulness of vaccination in general*. To assess belief in the usefulness or efficacy of vaccines, we used a 5-item scale (e.g., I believe that vaccines are a safe and reliable way to prevent the spread of disease; I believe that vaccines have negative effects that outweigh their positive effects in vaccination; Vaccines are rigorously tested in laboratories and would not be available to the public if they were not safe; The risk of injuring and killing children through vaccination outweighs the health benefits; Immunization is one of the most important contributions to public health; see 38]. Participants were asked to rate their responses on a 7-point scale ranging from 1 = Very strongly disagree to 7 = Very strongly agree. We carried out a PCA on the 5 items of the scale (KMO = .82 KMO per item >.80; χ^2^(10) = 592.24, *p* < .001). Parallel analysis [44] indicated that only one component (λ = 2.95) should be used, which explained 59.00% of the variance. All item saturations on this single component were greater than .54 (in absolute value). A confirmatory factor analysis indicated a correct fit of the data to a one-factor model (CFI = .980; TLI = .961; SRMR = .028; RMSEA = .085). The item scores were averaged to obtain a total score that represented perceived efficacy of vaccines. The higher the score, the more effective the vaccine is perceived to be by the stakeholders (α = .81).

*Political orientation*. We measured participants’ own political orientation using a 11-point scale that was anchored far left and far right [5].

*Conspiracy Beliefs*, *Subjective social class & Credibility of Science Scale*. We used the same measure as in the previous study (Study 1b).

### Results & discussion

In terms of the psychometric qualities of the credibility scale with respect to science, the results of this study were similar to those observed in Study 1a. The descriptive statistics of the different items indicated a relatively normal distribution of scores (see Table 5). The joint examination of the descriptive statistics of the overall score (see Table 5), the histogram representing the distribution of scores on the Credibility of Science Scale, and the quantile-quantile plot of scores on the same scale (see additional material, Figs 3 and 4 in S1 File), indicated that the distribution was also almost normal.

**Table 5:** Descriptive statistics of the items on the CoSS–Study 1C.

	M	SD	Min	Max	Skewness	Kurtosis
People trust scientists a lot more than they should	3.47	1.80	1.00	7.00	0.20	-1.14
People don’t realize just how flawed a lot of scientific research really is	4.55	1.60	1.00	7.00	-0.47	-0.47
A lot of scientific theories are dead wrong	3.32	1.64	1.00	7.00	0.47	-0.54
Sometimes I think we put too much faith in science	3.55	1.91	1.00	7.00	0.20	-1.21
Our society places too much emphasis on science	3.10	1.83	1.00	7.00	0.52	-0.82
I am concerned by the amount of influence that scientists have in society	3.03	1.76	1.00	7.00	0.57	-0.68
*CoSS*	3.50	1.41	1.00	7.00	0.19	-0.73

We carried out a PCA on the 6 items of the scale (KMO = .89 KMO per item >.88; χ^2^(15) = 1032.46, *p* < .001). Parallel analysis [44] indicated that only one component (λ = 3.85) should be used, which explained 64.25% of the variance. All item saturations on this single component were greater than .69. A confirmatory factor analysis indicated a correct fit of the data to a one-factor model (CFI = .980; TLI = .967; SRMR = .033; RMSEA = .083). Nevertheless, the value of the RMSEA was slightly too high, and an examination of the residue correlation matrix told us to add a correlation between the errors of items 2 and 3, thus the adjustment improved (CFI = .996; TLI = .992; SRMR = .017; RMSEA = .039). Finally, the internal consistency was satisfactory (α = .88). The item scores were averaged to obtain a total score that represented the credibility that participants gave to science. The higher the score, the more credibility participants gave to science.

An analysis of correlations highlighted three significant correlations (cf. Table 6). The first highlights the link between mistrust of science and intentions to be vaccinated against COVID-19: the more mistrustful participants are of science, the less willing they are to be vaccinated against the virus. The second highlights the link between mistrust of science and perceptions of vaccine effectiveness: the more skeptical participants are about science, the less effective they think vaccines are. The third one highlights the link between mistrust of science and conspiracy beliefs and indicates that the more participants adhere to conspiracy beliefs, the more they doubt the credibility of science. However, we did not observe any significant difference according to the sex of the participants (*p* = 0.55).

**Table 6:** Correlations between the different variables presented in Study 1C.

	r	p
Vaccination intentions COVID-19	-0.37	< .001
Effectiveness of vaccines	-0.54	< .001
Conspiracy Beliefs	0.53	< .001
Subjective social class	-0.06	.244
Political Orientation	-0.05	.342
Level of education	-0.09	.100
Age	0.04	.467

Note. Significant correlation coefficients are presented in bold.

We performed a regression analysis to predict the credibility given to science with the following variables: adhesion to conspiracy beliefs, political orientation, subjective social class, level of education and age (R^2^ = 0.30, *F*(5,312) = 26.84, *p* < .001). Results are presented in Table 7. Only the adhesion to conspiracy beliefs predicts positively the credibility accorded to science: the more participants adhere to conspiracy beliefs, the more distrustful they are of science.

**Table 7:** Regression analysis to predict credibility towards science (Study 1C).

**Predictor**	**β**	**SE**	**95% IC**	**t**	**p**
Intercept	1.32	0.39	[0.548;2.102]	3.36	< .001
Conspiracy Beliefs	0.85	0.07	[0.700;0.995]	11.27	< .001
Political Orientation	-0.05	0.03	[-0.103;0.003]	-1.87	0.062
Subjective social class	0.02	0.04	[-0.060;0.097]	0.46	0.645
Level of education	-0.01	0.04	[-0.091;0.060]	-0.40	0.687
Age	0.00	0.01	[-0.091;0.014]	0.40	0.692

The psychometric qualities of the scale were satisfactory. Beyond an almost normal distribution of scores, the French version of the scale showed good structural validity, good fidelity (homogeneity), and good criterion validity. In accordance with the results of previous work [4,20] we found a positive, and rather strong, correlation between adherence to a conspiracy mentality and the skepticism towards science. Similarly, the results show that the more confident participants are in science, the greater their acceptance of vaccination. Finally, the most important result seems to be the relationship between distrust of science and intentions to be vaccinated against COVID-19: indeed, the results show that the more confident participants are in science, the more likely they are to be vaccinated. These results thus reinforce the importance of considering the credibility of science in public health issues.

## Study 2

This study aimed to replicate previous results regarding the psychometric qualities of the scale. In addition, it also aimed to test the hypothesis of a link between the social representations of science and its level of perceived credibility. More specifically, we expected that the perceived credibility of science would contribute to the organization of the socio-representational field of this object. Finally, we expected a link between the attitude associated with the socio-representational field and the score on the perceived Credibility of Science Scale.

### Method

#### Population, procedure & measures

The study took the form of an online questionnaire distributed via social networks (on local self-help groups and local community groups). Data collection took place between 12 February and 16 March 2019. The participants were invited by a message published in city self-help Facebook groups. The invitation message mentioned that the study was about how to see the world, with no specific mention of science. Four hundred and four participants all participated freely and without compensation in this study (77.23% of women, *M*_*age*_ = 28.44, *SD* = 11.48, age range: 16–72). After applying the exclusion criteria (legal minimum age, participants who reported being distracted or disturbed, abnormally long or short time spent) the final sample consisted of 319 participants (79.6% of women, *M*_*age*_ = 29,98, *SD* = 11.55, age range: 18–72). After freely consenting to participate in the study, participants completed some socio-demographic characteristics (gender and age), then first completed a free association task [59–62]: participants had to produce the 4 words or expressions that came to mind when they thought of the inducer, namely "science". The 4 words or expressions produced were presented again to the participants who were then asked to judge whether they were positive or negative. Thus, were then asked to rate the attitude associated with each of their verbal production by stating whether it was positive or negative on a 7-point Likert scale ranging from -3 (*completely negative*) to +3 (*completely positive*) [63]. They then completed the same scale of credibility in science as in Studies 1b and 1b.

### Results and discussion

The descriptive statistics of the different items indicated a relatively normal distribution of scores (see Table 8).

**Table 8:** Descriptive statistics of the items on the CoSS–Study 2.

	M	SD	Min	Max	Skewness	Kurtosis
People trust scientists a lot more than they should	4.07	1.66	1.00	7.00	-0.21	-0.85
People don’t realize just how flawed a lot of scientific research really is	4.71	1.50	1.00	7.00	-0.50	-0.28
A lot of scientific theories are dead wrong	3.96	1.49	1.00	7.00	-0.11	-0.39
Sometimes I think we put too much faith in science	4.11	1.72	1.00	7.00	-0.20	-0.99
Our society places too much emphasis on science	3.58	1.75	1.00	7.00	0.31	-0.96
I am concerned by the amount of influence that scientists have in society	3.41	1.65	1.00	7.00	0.25	-0.86
*CoSS*	3.97	1.20	1.00	7.00	-0.18	-0.51

*Factor structure of the scale*. We performed a PCA on the 6 items of the scale (KMO = .85, KMO per item >.82; χ^2^(15) = 680.50, *p* < .001). A parallel analysis [44] indicated that only one component (λ = 3.28) should be used, which explained 54.79% of the variance. All item saturations on this single component were greater than .56. Confirmatory Factor Analysis indicated a correct fit of the data to a one-factor model (CFI = .969; TLI = .949; SRMR = .037; RMSEA = .084). As before, to obtain better adequacy indices, we added a correlation between items 2 and 3: the adjustment improved (CFI = .983; TLI = .969; SRMR = .027; RMSEA = .066). Finally, the internal consistency was satisfactory (α = .83). It should be noted that, in terms of psychometric qualities, the results observed in this study were comparable to the previous results.

*Age & Gender*. We do not observe a significant correlation between the age of the participants and the science credibility score (r = -.02, p = 0.75). Nevertheless, contrary to the results of previous studies, we observe a significant effect of the gender of the participants (t(317) = 2.13; p = 0.03, η^2^_p_ = .01). Women give more credibility to science (M = 4.05 SD = 1.20) than men (M = 3.66 SD = 1.20). Nevertheless, this effect is small.

*Social representation of Science*. The terms associated by the participants in the context of the task of free associations dealing with the "science" inducer were lemmatized [64,65]. On this occasion, terms sharing a common root were grouped together. Under this data processing phase, there are 260 different terms. The most common terms (cited by more than 10 participants) are presented in Table 9.

**Table 9:** Attitudes and average ranks associated with the terms most frequently associated with science by participants.

	N	Rank		Attitude			N	Rank		Attitude	
		M	SD	M	SD			M	SD	M	SD
research experience advancements medicine physical study discovery mathematics life chemistry laboratory human and social sciences biology knowledge nature human cosmos technology body	100 55 49 40 39 39 38 35 27 26 23 23 22 22 21 20 20 20 19	2.3000 2.5273 2.2653 2.5750 2.2564 2.3590 2.8421 2.1714 1.8148 2.5769 2.7391 2.2609 2.1364 2.5909 2.1905 2.3000 2.7500 2.6000 1.3684	1.1677 1.0157 1.0363 0.9578 1.2294 1.1118 1.1746 1.2244 0.9214 0.9454 1.0539 0.9638 0.9409 1.1816 1.1233 0.9787 1.1642 1.0954 0.5973	2.2900 1.1818 2.0408 2.5250 0.7436 1.6410 2.7895 0.6000 2.2222 0.9231 0.9565 2.6522 1.4545 2.5455 1.9048 1.6000 1.2000 1.8000 2.0526	1.1037 1.6340 1.3687 0.8767 1.4458 1.2028 0.4741 1.8020 1.5275 1.5728 1.3307 0.7141 1.5653 0.8004 1.6095 1.3917 1.7045 1.3219 1.2236	scientific knowledge base molecule researcher evolution Earth future intelligence Einstein healthiness sickness accurate life sciences animals interesting future	18 17 17 17 16 15 15 13 13 13 13 12 11 11 10 10	2.6111 2.8824 2.4118 2.7059 2.5625 2.2000 2.7333 2.2308 2.8462 2.4615 2.9331 1.7500 2.5455 2.5455 2.6000 3.5000	1.2897 1.1663 1.0641 1.0467 0.9639 0.8619 1.3345 1.3009 1.0682 0.8771 0.7596 1.2154 1.2136 1.0357 1.0750 0.9718	1.5000 2.8235 0.8824 2.1176 2.0000 1.3333 1.6000 2.3846 2.3077 2.2308 -0.1538 0.9167 1.1818 0.6364 1.7000 2.1000	1.3394 0.3930 1.2690 0.9926 0.7303 1.9518 1.8048 0.7679 0.9473 1.0127 2.7033 1.4434 1.3280 2.3779 1.4181 1.1972

These seem to reflect a rather positive perception of science. Indeed, none of the terms presented seem to reflect a critical view of this object. On the contrary, the terms presented underline in some cases a favorable positioning (e.g., interesting, future) or, more often, a definition/description of science through the concepts associated with it (e.g., research, study, discovery, etc.) or its fields of application (e.g., Human and Social Sciences, biology, mathematics, etc.).

*Attitude associated with the socio-representative field*. The attitudinal positioning associated with all the terms mentioned by the participants (*M =* 1.84; *SD* = 1.10) confirms the observation of a rather positive representation of science. Moreover, as expected, we observe a significant relationship between the attitude associated with the socio-representational field and the scores on the science credibility scale (*r =* -.29, *p* < .001): the more distrustful participants are of science, the more they associate negative words.

*Correspondence Factor Analysis*. Finally, we carried out a Correspondence Factor Analysis (Corr.F.A.). This factor analysis allowed us to highlight the differences in the frequency of association of terms derived from the free association task according to the modalities of the independent variables. It also, on the one hand, highlighted the variables that have the greatest impact in the differentiation of the content evoked and, on the other hand, provided a summary of the data indicating the correspondences between the modalities of the independent variables and terms associated by participants [63,66].

**Fig 1:**
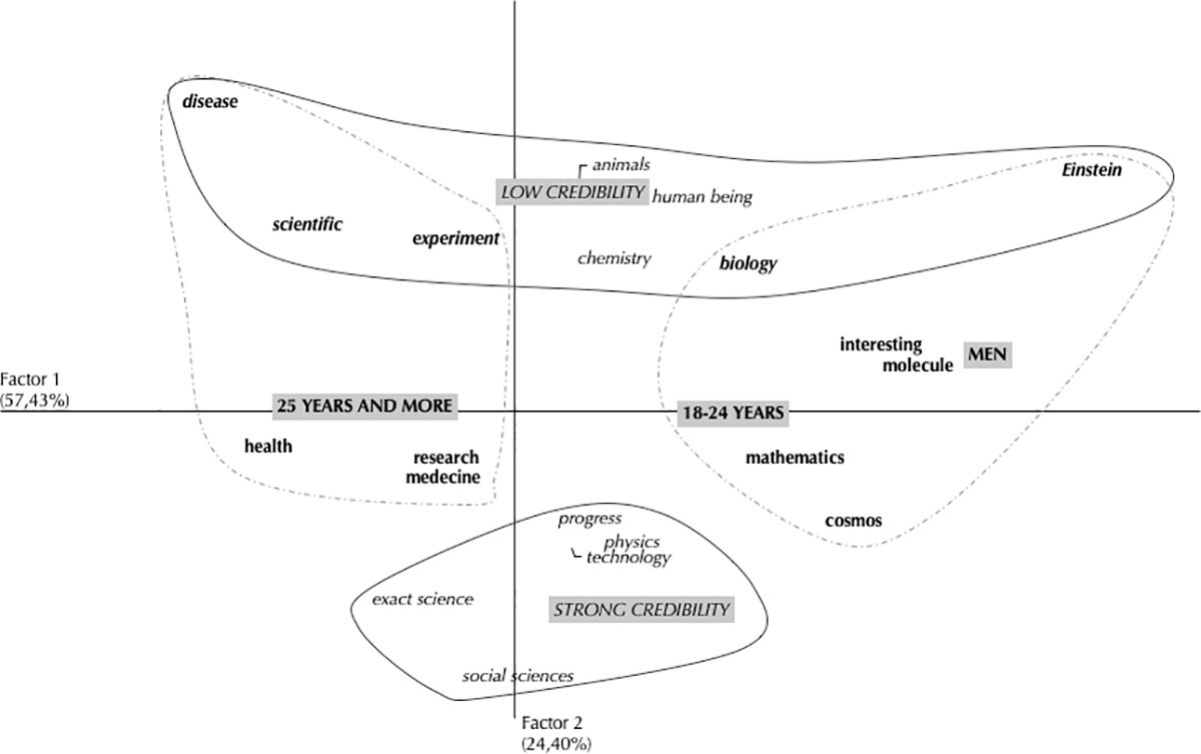
Results of the factorial analysis of correspondences related to science-related evocations. Grayed frames refer to the independent variables. “Independent variables” contribute to the formation of Factor 1; “Independent variables” refers to the independent variables which contribute to the formation of Factor 2. “Observations” refers to the observations which contribute to the formation of Factor 1; “Observations” refers to the observations which contribute to the formation of Factor 2; “Observations” refers to the observations which contribute to the formation of both Factors 1 and 2.

The analysis was thus conducted by mobilizing terms with a frequency greater than or equal to 10 (see Table 9), i.e., 859 verbal productions representing 76.22% of the corpus which consists of 1127 productions, excluding isolated terms. The Corr.F.A. was conducted on the basis of a contingency table containing the 35 selected verbal productions and the various independent variables dichotomized on the basis of the median (i.e., the level of credibility of science, age and gender). The Corr.F.A. highlighted two factors that accounted for 81.84% of the table’s inertia (Factor 1–57.43%; Factor 2–24.40%; see Fig 1). Only the modalities of the variables and the types of responses contributing to the construction of the factors were retained. By construction, the sum of the contributions by factor (CF) of the variable’s modalities or response types being equal to 1, we retained the modalities or types whose contribution by factor was greater than the average contribution (i.e., 1/6–0.167 for the variable modalities, and 1/32–0.028 for the response types) [67]. Thus, the modalities of the variables that contribute to the construction of factor 1, re "Age 18 to 24 years" and "Age over 25": CF (Age 18–24) -0.17—CF (Age Over 25) -0.18, for a total contribution of 35.19% to the formation of Factor 1. Factor 2 is constructed by the modalities of the variables "low science credibility" and "high science credibility": CF (low credibility of science) = 0.51 + CF (strong credibility towards science) = 0.47 or a total contribution of 98.27%.

Concerning Factor 1, there is mainly an opposition between the modalities related to the age of the participants. It appears that older participants seem to associate terms more closely with health-related concerns (e.g., health, medicine, disease). However, from the point of view of our assumptions, the second factor is more decisive. Indeed, as expected, the opposition between the low and high credibility of science on this second factor reflects the link between credibility scale scores and the socio-representational field. In terms of content, it was noted that participants with the highest ranking on the credibility scale of science evoked terms that were more focused on a vision of science that combined modernity (e.g., progress, technology), exact sciences and physics. On the other hand, participants with a weaker position on the credibility scale of science put more emphasis on life sciences (e.g., human being, animals, biology) as well as terms that can be considered more stereotypical (e.g., scientist, Einstein, experiment).

## General discussion

The objective of this article was on the one hand to validate the CoSS in French and on the other hand to highlight the links between the different levels of credibility and the social representations of science. In order to do so, we conducted three empirical studies which aimed to study the psychometric qualities of the scale and to show that different socio-representational contents were associated with different levels of credibility.

Studies 1a and 1b demonstrated the good structural validity, the good fidelity (homogeneity and temporal stability), and the good criterion validity of the French version of the scale. In accordance with Hartman et al. [20] we found a positive, and rather strong, correlation between adherence to a conspiracy mentality and the mistrust of science. We also showed that the more participants perceive themselves as belonging to a high social class, the more credibility they give to science. In study 2, we observed the same psychometric qualities of the French version of the scale.

These studies also allowed us to observe results which indicate skepticism toward science among a section of the participants. Indeed, while in Study 1a the participants declared themselves, on average, rather confident in science, in Study 1b the average was closer to the center of the scale. An examination of the distributions also showed a fairly large proportion of participants with a low confidence in science. However, this positioning did not particularly appear in regard to the social representation of science in Study 3. Indeed, regarding the responses given by the participants (i.e., the words resulting from the association task) and their attitude associated with the socio-representative field, it appeared that they had a rather positive representation of science. Nevertheless, as for the responses related to the CoSS, a certain part of our sample may share a negative representation of science. Thus, the Corr.F.A. allowed us to highlight the content associated by the participants who had a high score on the CoSS (i.e., participants who attribute a poor credibility to science).

One of the limitations of our studies is that they were all carried out on convenient samples. This method of collecting data makes it possible to have quick and easy access to a large group of participants, who are relatively varied, but not representative of the French parent population. Certain aspects of the population therefore deserve to be discussed. The age of the participants is negatively associated with the credibility score of science only in study 1a. We conducted a small-scale meta-analysis on the results of the four studies presented in order to estimate the average correlation between age and science credibility. This analysis reveals the following average correlation: *r* = -.03 *p* = .32 CI[-0.096;0.031]. Thus, the age of the participants would not be related to the level of credibility of the science. We also conducted a meta-analysis on gender results. The average difference between the two groups was *g* = -0.01, (*p* = 0.946, 95% CI[-0.23, 0.22]). These results suggest that there is no effect of participants’ gender on the level of credibility they attach to science. Nevertheless, these results would deserve to be confirmed on a representative sample of the French population.

Regardless, these results are in line with the general dynamic observed in other countries. Indeed, as we mentioned, this phenomenon is not specific to France. Thus, in the United Kingdom the House of Lords Select Committee on Science and Technology stated that “society’s relationship with science is in a critical phase characterized by public unease, mistrust and occasional outright hostility” [68]. In Spain, Luján and Todt observed that according to Spanish citizens, scientists can be influenced by economic interests [69].

This negative positioning against science is not without consequences. Indeed, it can negatively affect the public funding and even the implementation of decisive research programs [70]. In this context, one can note the relationship between attitudes toward science and positionings in favor of the public funding of research programs [71]. Moreover, as we mentioned, this skepticism regarding science may also constitute one of the possible causes of detrimental health or environment related behaviors through disagreement over the efficiency of vaccines or the existence of global warming.

Our results also raise the question of possible means of intervention to promote a better perception of science. In this context, in 2001 the European Commission launched the Science and Society Action Plan [72]. This plan aimed to give European citizens a better grasp of science. However, this plan was based on the postulate according to which negative attitudes toward science mainly come from a lack of understanding of this object. Yet, it appears that this phenomenon is the consequence of several factors. Thus, negative attitudes toward science would rather result from the interaction between a lack of understanding of this object and of the links, ways of control and regulation that exist between scientists and institutions [68]. Thus, beyond the perception of science, which can be positive, it is also the knowledge of the complex relations with institutional and economic issues which may lead people to mistrust science [69]. In that regard, aside from understanding and trust in science, trust in companies and institutions may also be a lever to promote a better perception of the credibility of science and lower the perceived risks associated with technological scientific developments [73]. This is all the truer as some of the arguments against vaccination are based on a postulated influence of scientists by economic interests and pharmaceutical companies. Moreover, at least in France, these companies are also sometimes accused, by people opposed to vaccination, of influencing the political agenda and public health measures regarding vaccination. In this perspective, the relationship that we observed between the responses regarding the CoSS and the Generic Conspiracist Beliefs Scale seem to be in line with this phenomenon. Thus, the validation of the scale of credibility towards science is a major issue for future studies that will attempt to explain attitudes of rejection or mistrust towards science and towards important societal issues (e.g., vaccination, global warming) but also to try to develop actions to promote science that will aim to change pro-environmental and preventive health behaviors.

## Supporting information

S1 File (DOCX)
