# Supplementary material for: Skepticism and defiance: Assessing credibility and representations of science
Source: PLoS One. 2021 Sep 1;16(9):e0250823. doi: 10.1371/journal.pone.0250823 (PMC8409661; doi:10.1371/journal.pone.0250823)
Supplement: S1 File — (DOCX) [file pone.0250823.s001.docx]

**Additional Material**


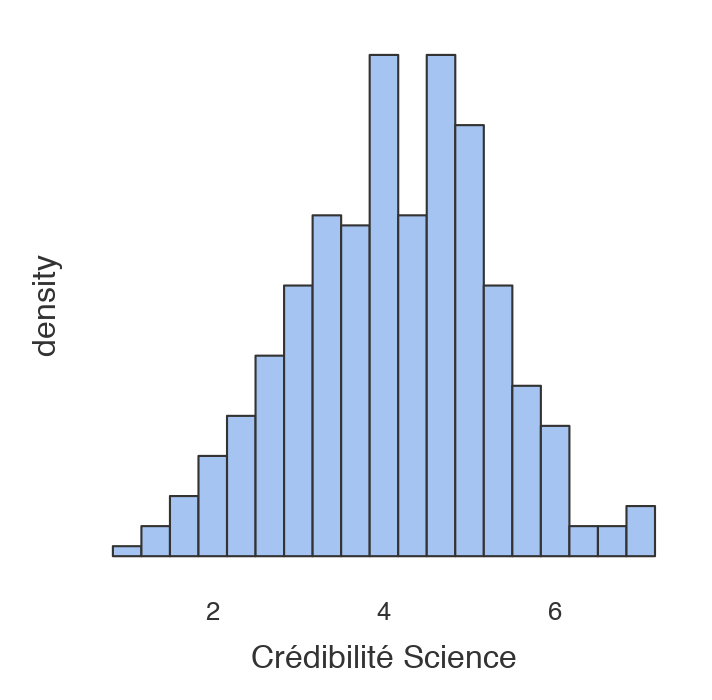


Figure 1: Histogram representing the distribution of scores on the Credibility of Science Scale.


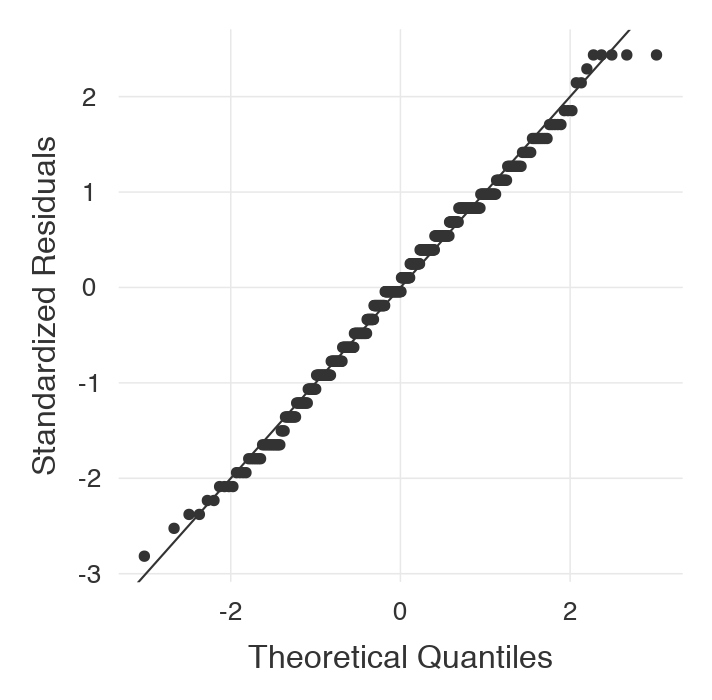


Figure 2. Quantile-Quantile Diagram of Credibility of Science Scale Scores.


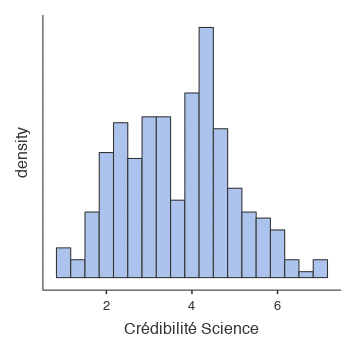


Figure 3: Histogram representing the distribution of scores on the Credibility of Science Scale.


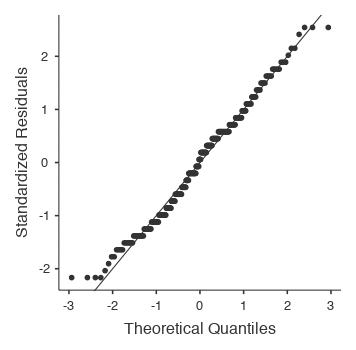


Figure 4. Quantile-Quantile Diagram of Credibility of Science Scale Scores.

**Appendix 1**

Version FR

**Votre façon de voir la communauté scientifique et son travail.**

Nous allons vous présenter une série d’affirmations concernant les scientifiques et la communauté scientifique. Merci d’indiquer à quel point chaque affirmation décrit ce que vous pensez – c’est-à-dire, à quel point vous êtes d’accord ou en désaccord avec chaque affirmation.

Merci de noter que ces affirmations se centrent sur votre impression générale concernant la communauté scientifique actuelle, ses méthodes et ses conclusions.

Certaines affirmations peuvent vous sembler répétitives ou redondantes, mais même si cela vous semble être le cas, merci de prendre le temps d’évaluer chacune d’elle séparément.

| Les gens font confiance dans les scientifiques plus qu’ils ne devraient |
| --- |
| Les gens ne réalisent pas à quel point de nombreuses recherches scientifiques comportent de sérieux défauts |
| Un grand nombre de théories scientifiques sont complètement fausses |
| Parfois, je pense que nous accordons trop de confiance à la science. |
| Notre société accorde une place beaucoup trop importante à la science |
| Je suis inquièt.e de l'influence importante qu'ont les scientifiques dans la société |
